# Baltimore Clinical Society

**Published:** 1878-05

**Authors:** 


### MEETINGS OF BALTIMORE CLINICAL SOCIETY.

Regular meetings of the Clinical Sooiety have been held the last month, with full attendance of members, who have manifested an unusual degree of interest in the proceedings.

Dr. Michael showed specimens of anchylosis of the knee and hip, from subjects brought to the dissecting room. The specimens were prepared by dividing them with a fine saw, thus beautifully showing complete ossification of the joints.

Dr. Theobald showed two foreign bodies removed from the external auditory meatus of children about six years old. One was a black-eyed red bean, the other a coffee grain. Attempts to remove them with syringes and forceps had resulted in pushing them still further in. Dr. T. deprecated the use of forceps in such cases, but recommended a probe bent at a proper angle. By gently pushing the bean from behind, it could be rolled over and over until taken out. An anæsthetic must be taken to insure success. Dr. T. also related a case of glioma of the retina in a boy six years old, which was not apparent on superficial inspection, but was disclosed by the ophthalmoscope. The point of interest was the age of patient.

Dr. Tiffany called attention to the use of wire gauze, introduced by him to lighten and strengthen the plaster of Paris splints. Common wire gauze, such as is used for fly nets is cut into slips and placed up and down the limb on three sides. The plaster fills the meshes of the gauze and forms a hard, compact mass, much lighter than the common splint and of greater strength. It had been used with great success. If the gauze be not at hand, a layer of iron filings dusted over the plaster before it dries, hardens it, and does not necessitate the use of so great a quantity.

Dr. Tiffany also related two cases of popliteal aneurism treated by pressure.

The first case, a patient æt. 35, who five years ago had a chancre, and went through the various stages of syphilis, on the 19th September, 1876, noticed swelling in his left ham, with pain.

Was found to be two inches larger than the right, and on the 11th, it was possible to make a diagnosis of popliteal aneurism. Two horse-shoe tourniquets were applied alternately, a sea-saw motion being kept up between them as pain in the one or the other became too great. By Christmas there was great improvement. Then the tumor became larger, and flexion was made of the knee. Between the 10th and 18th February, great improvement took place, and on the 22nd no pulsation could be felt, while the tumor hardened.

Veratrum viride and morphia were used in sufficient quantities to lessen the pulse and pain. The aneurism was entirely cured. A noticeable point was the enlargement of all the arteries about the knee-joint, collateral circulation being thus set up, which confirmed the cure.

The second case was that of a very old negro who complained of pain in his right ham. Examination showed enlargement, pulsation aneurismal bruit. Forcible flexion was kept up thirteen hours. The patient was so aged as evidently to be dying of old age, so the prognosis was unfavorable. The tumor disappeared considerably before death, which occurred three or four weeks afterwards. *Post mortem* showed a double aneurism, the upper one being entirely filled with a clot which thoroughly occluded that part of the artery, while the lower one had been ruptured, and contained a clot also.

Dr. T. R. Brown related a case of popliteal aneurism treated by flexion for eighteen days without success. Then digital pressure and tourniquets were tried. Under this treatment the symptoms subsided, and the patient was doing well, till suddenly, after a week, the knee swelled, the patient became delirious, and ten days after died of phlebitis.

Dr. B. also showed a specimen of malignant disease of the cheek, from a patient who was ninety years old when it first appeared. The age of patient was the point of interest. Also specimens of atheromatous arteries, also two dorsal vertebræ, from the spinal column of Captain Conway, in which was imbedded a minnie ball. The captain was shot while dredging for oysters. When admitted into the hospital, his pulse was 120, temperature 101, and he was overcome by continuous rigors. He could not move his feet, but there appeared to be hyperæsthesia of the skin. Urine retained. *Post mortem* showed hypostatic congestion of lungs, innumerable abscesses of integument, and also large abscess of liver and cystitis, the spleen containing pus. Patient died of pyæmia. The bullet penetrated the spinal column on the right side, between the eleventh and twelfth dorsal vertebræ, and the point protruded into the spinal cord, injuring the cord.

Dr. Tiffany related a similar case of a sailor falling twenty-four feet over a railing of a vessel on his back. The patient died of pyæmia, with symptoms same as in the previous case. *Post mortem* showed no injury to the spinal column, but diffluence of the cord opposite the point struck.

Dr. Michael related a case of a soldier in the late war who was struck by a ball in the sacrum, and is still living. There is paralysis of lower limbs, retention of urine, and recurrence of bed-sores as soon as any pressure is allowed.

Dr. Tiffany, at a later meeting in March, exhibited: 1st. Malignant disease of testicles, which appeared eleven months before operation, increased to a large size, pushing aside the penis and other testicle. Another physician had tapped it twice for hydrocele. It was a small-cell sarcoma. 2d. Caries of Ilium. There had been a small opening over the left buttock for four years. It was a question whether disease started below in the acetabulum, or above and worked its way downwards. 3d. Fracture with non-union of Scaphoid bone. Patient had died of disease, a fracture of lower arm being made out before death. Upon examination, no sign of a fracture was found excepting the Scaphoid bone, which was in three pieces. Fourteen years before, the patient had fallen forward on his wrist and hand, causing the fracture. False joints had been formed between the three pieces, and the surfaces rubbed as smooth as polished ivory. 4th. Epithelioma capitis, which appeared three years before death, and increased rapidly, covering the greater part of one side of the scalp. There was ho glandular enlargement elsewhere.

Dr. Coskery—a case of gangrene following burn, with occlusion of axillary artery by thrombus.

Dr. Hill—a case of typhoenteritis, with perforation of vermiform appendix. Patient, a German, ate a large quantity of dried apples and cheese, after which was taken down with pain and cramps, getting continually worse. Diagnosis made of foreign matter in appendix. *Post mortem* showed appendix perforated and enlarged, and large abscess, containing quart of pus, extending as far up as diaphragm.

Dr. Atkinson—a case of papillary epithelioma occurring on the thumb, which he had removed with a curette. The result was astonishing, the wound healed so quickly. The ease and delicacy with which the diseased warts were scraped away with the curette made this instrument a valuable one in all such cases.

Dr. Rohe had used the curette with the same success, and extolled the results. Dr. R. also asked the society if any of the members had seen cases of leprosy in Baltimore. The disease is on the increase in the Northwest and among the Chinese in California, and is generally more common than it is usually thought to be. If it be contagious, it becomes an important subject, and he would thank any physician who would report cases to him.

Papers were read on "Ozena," by Dr. Hartman, and on "Latent" Vaginitis, by Dr. B. B. Browne.—*Maryland Medical Journal.*

